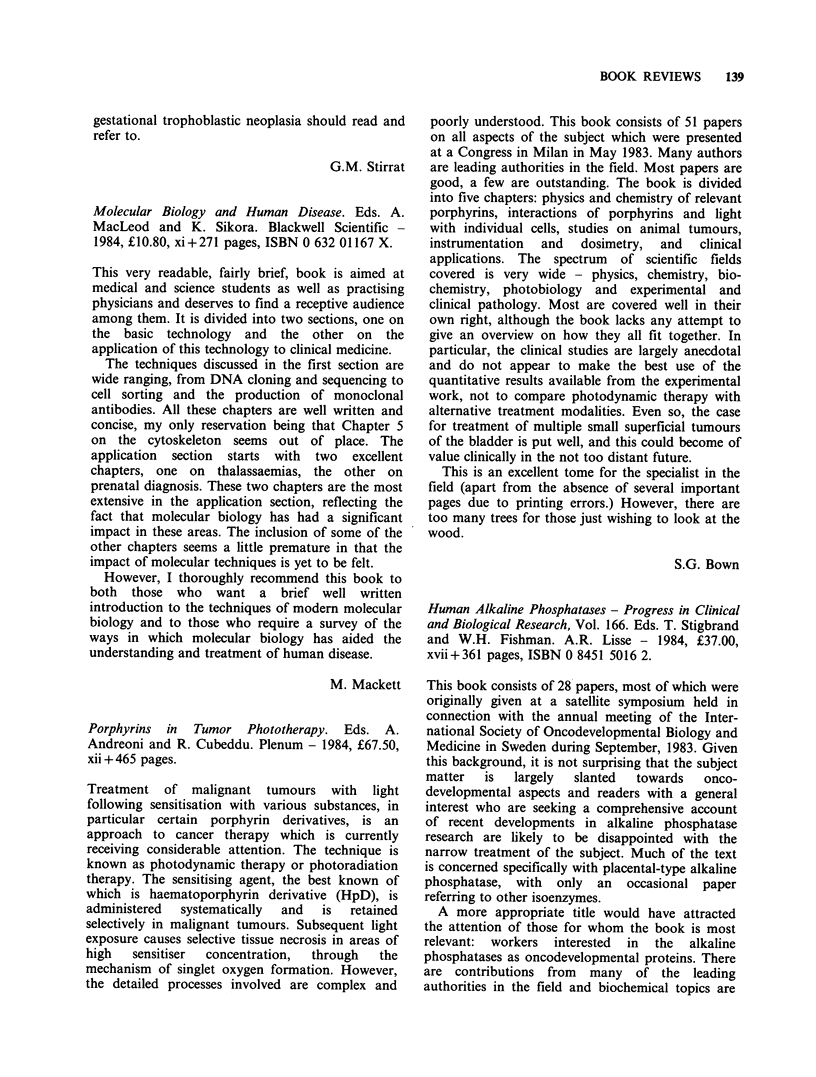# Porphyrins in Tumor Phototherapy

**Published:** 1985-07

**Authors:** S.G. Bown


*Porphyrins in Tumor Phototherapy*. Eds. A. Andreoni and R. Cubeddu. Plenum – 1984, £67.50, xii+465 pages.

Treatment of malignant tumours with light following sensitisation with various substances, in particular certain porphyrin derivatives, is an approach to cancer therapy which is currently receiving considerable attention. The technique is known as photodynamic therapy or photoradiation therapy. The sensitising agent, the best known of which is haematoporphyrin derivative (HpD), is administered systematically and is retained selectively in malignant tumours. Subsequent light exposure causes selective tissue necrosis in areas of high sensitiser concentration, through the mechanism of singlet oxygen formation. However, the detailed processes involved are complex and poorly understood. This book consists of 51 papers on all aspects of the subject which were presented at a Congress in Milan in May 1983. Many authors are leading authorities in the field. Most papers are good, a few are outstanding. The book is divided into five chapters: physics and chemistry of relevant porphyrins, interactions of porphyrins and light with individual cells, studies on animal tumours, instrumentation and dosimetry, and clinical applications. The spectrum of scientific fields covered is very wide – physics, chemistry, biochemistry, photobiology and experimental and clinical pathology. Most are covered well in their own right, although the book lacks any attempt to give an overview on how they all fit together. In particular, the clinical studies are largely anecdotal and do not appear to make the best use of the quantitative results available from the experimental work, not to compare photodynamic therapy with alternative treatment modalities. Even so, the case for treatment of multiple small superficial tumours of the bladder is put well, and this could become of value clinically in the not too distant future.

This is an excellent tome for the specialist in the field (apart from the absence of several important pages due to printing errors.) However, there are too many trees for those just wishing to look at the wood.

S.G. Bown